# A Manual of the Diagnosis and Treatment of the Diseases of the Eye

**Published:** 1900-02

**Authors:** 


### BOOK REVIEWS, PAMPHLETS, EXCHANGES.

### BOOK REVIEWS.

[Contributions solicited for review.]

A Manual of the Diagnosis and Treatment of the Diseases of the Eye. By Edward Jackson, A.M., M.D. Emeritus Professor of Diseases of the Eye in the Philadelphia Polyclinic. Member of the American Ophthalmological Society, etc. Published by W. B. Saunders, Philadelphia. Price $2.50, net.

There are at present before the profession several small works on Diseases of the Eye, such as Nettleship, Swanzy, Mittendorf, in addition to the numerous larger ones classed as text-books. It is rather difficult to see how any one could have the temerity to bring forth yet another one. Dr. Jackson in this volume has given us a clear and concise treatment of all the subjects and laid out lines which are safe to follow. The author is recognized for his eminently scientific and practical work on refraction, so that it is not surprising to find the chapters dealing with this portion of the subject unusually clear. The arrangement of the various chapters is very similar to that found in other works on ophthalmology. The illustrations are good. Like all the rest of such small books, it no doubt has a field of its own, and yet it is hard to see how so many books of such marked similarity can pay even the author and publisher. The general make-up of the work is good. R.

